# Prediction and Identification of Potential Immunodominant Epitopes in Glycoproteins B, C, E, G, and I of Herpes Simplex Virus Type 2

**DOI:** 10.1155/2012/205313

**Published:** 2012-05-09

**Authors:** Mingjie Pan, Xingsheng Wang, Jianmin Liao, Dengke Yin, Suqin Li, Ying Pan, Yao Wang, Guangyan Xie, Shumin Zhang, Yuexi Li

**Affiliations:** ^1^Department of Biochemistry and Molecular Biology, School of Preclinical Medicine, Nanjing Medical University, Nanjing 210029, China; ^2^Department of Medical and Pharmaceutical Biotechnology, Huadong Research Institute for Medicine and Biotechniques, Nanjing 210002, China; ^3^Department of Pharmacy, Anhui University of Traditional Chinese Medicine, Hefei 230038, China; ^4^School of Life Science and Technology, China Pharmaceutical University, Nanjing 210009, China; ^5^Laboratory of serology, National Institutes for Food and Drug Control, Beijing 100050, China

## Abstract

Twenty B candidate epitopes of glycoproteins B (gB2), C (gC2), E (gE2), G (gG2), and I (gI2) of herpes simplex virus type 2 (HSV-2) were predicted using DNAstar, Biosun, and Antheprot methods combined with the polynomial method. Subsequently, the biological functions of the peptides were tested via experiments *in vitro*. Among the 20 epitope peptides, 17 could react with the antisera to the corresponding parent proteins in the EIA tests. In particular, five peptides, namely, gB2_466–473_ (EQDRKPRN), gC2_216–223_ (GRTDRPSA), gE2_483–491_ (DPPERPDSP), gG2_572–579_ (EPPDDDDS), and gI2_286-295_ (CRRRYRRPRG) had strong reaction with the antisera. All conjugates of the five peptides with the carrier protein BSA could stimulate mice into producing antibodies. The antisera to these peptides reacted strongly with the corresponding parent glycoproteins during the Western Blot tests, and the peptides reacted strongly with the antibodies against the parent glycoproteins during the EIA tests. The antisera against the five peptides could neutralize HSV-2 infection *in vitro*, which has not been reported until now. These results suggest that the immunodominant epitopes screened using software algorithms may be used for virus diagnosis and vaccine design against HSV-2.

## 1. Introduction

Herpes simplex virus type 2 (HSV-2) mainly causes genital infections [1]. HSV-2 glycoproteins are structural components of the virion envelope and have been implicated in virus-induced alterations of mammalian cells [2, 3]. Moreover, HSV-2 glycoproteins are expressed in infected cell plasma membranes and act as major antigenic stimuli for the cellular and humoral responses of a host [2, 3]. Some virion glycoproteins, such as glycoproteins B (gB2), C (gC2), and E (gE2), have been described [4]. Glycoproteins G (gG2) and I (gI2) and gB2, gC2, and gE2 apparently play major roles in immune responses to HSV-2. Evidence support the following suggestions: (i) the purified gB2, gC2, gE2, gG2, and gI2 of HSV type 1 (oral, HSV-1) or HSV-2 (genital) stimulated high titers of type-common virus-neutralizing antibodies [5, 6]; (ii) passive immunizations with the monoclonal antibodies against gB2, gC2, gE2, gG2, and gI2 protected mice from the infection using a lethal dose of HSV [7–9]; (iii) gB2, gC2, gE2, gG2, and gI2 mediated antibody-dependent, complement-mediated cytotoxicity and antibody-dependent, cell-mediated cytotoxicity [7, 10, 11]; (iv) the purified gB2, gC2, gE2, gG2, and gI2 were able to protect mice against lethal infection with either HSV-1 or HSV-2.

We have recently predicted and identified the epitopes of gD2 involved in its biological and immunological activities using a panel of gD2-specific antibodies that recognized four separate antigenic epitopes on the molecule. Some epitopes are type-specific and have strong virus-neutralizing activity* in vitro*. In the current study, 20 immunodominant epitopes from five proteins of HSV-2, namely, gB2, gC2, gE2, gG2, and gI2, were predicted and validated via experiments. Five of the 20 epitopes had strong antigenicity and immunogenicity, and thus they could protect the mice from HSV-2 infection.

## 2. Materials and Methods

### 2.1. General Materials and Animals

 Medium199 (Applichem) contained 5% newborn calf serum, antibiotics, and 20 mM N-2-hydroxyethylpiperazine-N′-2-ethanesulfonic acid (HEPES). Bio-Rad miniprotean cell (Hercules, CA, USA), nitrocellulose membrane (Amersham, Arlington Heights, IL, USA), peroxidase-labeled goat anti-mice immunoglobulin G (Boster, China), and ECL detection system (Amersham, Pharmacia Biotech, Uppsala, SE, Sweden) were used in the current experiment. Antibodies to gB2, gC2, gE2, gG2, and gI2 were bought from ABcam Company. The mice (KM, China, 4–6 weeks old, female) were purchased from the Animal Centre in Anhui University of Traditional Chinese Medicine.

### 2.2. Epitope Prediction and Synthesis

The B-cell antigenic epitope analysis on six glycoproteins, namely, gB2 (AAA60540.1 gI2: 6234000), gC2 (AAA66442.1 GI: 330266), gE2 (ABU45439.1 gI2: 156072176), gG2 (ABI32310.1 GI: 113200389), and gI2 (ACA28832.1 GI: 168805625) from HSV-2, was based on three related software algorithms, namely, Biosun, DNAstar, and Antheprot. B-cell epitopes were predicted by referring to the parameters, such as *β*-turn occurrence, hydrophilicity, surface probability, and flexibility, which have been shown to be indicative of potentially antigenic regions. The peptides comprising Asn-X-Thr/Ser sequences (glycosylation sites), proposed membrane-spanning regions, and C-terminal membrane-anchor sequences were excluded. The peptides showing common results from the software algorithms above were selected as candidate epitopes. The antigenic, surface-located regions (B-cell epitope) of the primary structure of the glycoproteins from HSV-2 should be predicted to generate the antibodies that might be reactive with the glycoproteins and possibly neutralize viral infectivity [12–14]. Some nonepitope peptides confirmed using EIA tests were selected as the controls. The Jinsite Company (China) chemically synthesized the screened epitope peptides.

### 2.3. Antigenicity Analysis Using EIA Tests

 The antigenicity of the predicted epitopes was determined using EIA with the antibodies to the corresponding parent glycoproteins (gB2, gC2, gE2, gG2, and gI2). 96-well plates were coated with free peptides in 100 *μ*L (2.5 *μ*g/mL) of 50 mM sodium bicarbonate buffer/0.6 M NaCl (pH value of 9.6) for each well at 4°C overnight. The next day, 130 *μ*L of blocking solution (1 × PBS, 1% bovine serum albumin) was added into each well after washing the plates five times with PBST at 4°C overnight. The antibodies of the five proteins gB2, gC2, gE2, gG2, and gI2 were diluted using 1 : 100 and then a twofold serial dilution. Consequently, 100 *μ*L was added into each well and then incubated at 37°C for 1 h. The plates were washed five times with PBST. Subsequently, 100 *μ*L of 1 : 10000 diluted peroxidase-labeled goat anti-mice immunoglobulin G (Boster) was added into each well and then incubated at 37°C for 1 h. All antibodies or antisera were diluted with phosphate-buffered saline (PBS) supplemented with 0.2 M NaCl and 0.3% Tween 80. The plates were washed five times with PBST, and the color was developed by adding 100 *μ*L of TMB substrate, which was a solution of peroxide (0.006%, vol/vol) and o-phenylenediamine (0.02%, wt/vol) in 2% methanol-50 mM sodium phosphate buffer (pH value of 5.6), incubated at 37°C for 10 min in the dark. The reaction was stopped by adding 50 *μ*L of 2 M H_2_SO_4_. The A*_450_* value was then measured using a Titertek Multiscan spectrophotometer (Titertek, Helsinki, Finland).

### 2.4. Conjugation of Peptides to Carrier BSA

Conjugation of peptides to carrier BSA was performed according to the reported methods [15]. After the conjugation reaction, the samples were dialyzed against PBS (pH value of 7.4). The molar peptide/BSA ratio in the various conjugates was determined via the amino acid analyses on the peptide-carrier conjugate and the untreated BSA. The molar peptide/BSA ratios were 5 to 10. Peptide carrier conjugates were stored at −20°C.

### 2.5. Mice Immunization and Antisera Preparation

Twenty mice were divided into two groups with 10 mice each. Approximately, 4 *μ*g of peptide-BSA was then emulsified in complete Freund's adjuvant and was injected subcutaneously for the first injection of each mouse. Approximately 2 *μ*g of the purified peptide-BSA emulsified in incomplete Freund's adjuvant was injected subcutaneously after 14 days for the second injection. The third injection was similar to the second injection. The PBS, as a control, was injected using the same above-mentioned procedure. Blood samples were collected 14 days after the third immunization and were then stored at −20°C.

### 2.6. Peptide Immunogenicity Detection Using EIA

Peptide-BSA-specific IgG antibodies in the antisera were determined using an endpoint EIA with the peptide-BSA as antigens, as described previously [16]. The titers were expressed as the reciprocals of the highest dilution of the sera with ratio values of 2.1. Moreover, proteins gB2, gC2, gE2, gG2, and gI2 were used as antigens for detecting the antisera using EIA.

### 2.7. Peptide Specificity Analysis Using Western Blot

Approximately 10 *μ*L of peptides-BSA conjugation was separated using 10% SDS-PAGE with a Bio-Rad miniprotean cell (Hercules, CA, USA) and then transferred to a strip of nitrocellulose membrane (Amersham, Arlington Heights, IL, USA). The nitrocellulose strip was blocked using a blocking buffer (5% skim milk in 20 mM Tris-HCl, pH 7.5, with 500 mM NaCl, 0.05% Tween 20) and was then shaken for 2 h at 37°C or overnight at 4°C. Subsequently, the strip was incubated with the antiserum diluted 1 : 50–1 : 10000 using a blocking buffer and was then shaken for 2 h at 37°C or overnight at 4°C. The membrane was then washed for 3 min to 5 min with PBST five times. The HRP-labeled goat anti-mouse antibodies (Boster, China) at 1 : 10000 dilution was added into the strip and was incubated at 37°C for 1 h. The membrane was washed 3 min to 5 min with PBST five times, and then the luminescence substrate was added, exposing an X-ray film using an ECL detection system (Amersham, Pharmacia Biotech, Uppsala, SE, Sweden) in a dark room.

### 2.8. Virus Neutralization Test

The antisera for the neutralization of HSV-2 (strain Sav) *in vitro* were assayed using the 50% plaque reduction assay [17]. The antisera were inactivated at 56°C for 30 min to inactivate the complements. The antisera were diluted using 1 : 4, followed by twofold serial dilutions, and were then incubated with an equal volume of HSV-2 (150 *μ*L, 600 PFU). HSV-2 viruses and the sera were diluted with medium 199 (Applichem), which contained 5% newborn calf serum, antibiotics, and 20 mM N-2-hydroxyethylpiperazine-N′-2-ethanesulfonic acid (HEPES). Approximately 100 *μ*L of the incubation mixture was plated into the monolayers of the Vero cells grown in 6-well trays for each well after 2 h incubation at 37°C. An overlay medium containing 0.5% methylcellulose was then added into the mixture after 1 h adsorption at 37°C. The cells were fixed and then stained with Giemsa solution after 2-3 days of storage at 37°C. Plaques were counted, and the neutralization titer was calculated as the reciprocal of the serum dilution times yielding a 50% plaque reduction.

### 2.9. Animal Protection Experiments

Sixty mice were randomly divided into six groups with 10 mice each. Five groups were immunized with the five epitopes, respectively, and the last group was immunized with PBS as the control. Each mouse was subcutaneously immunized thrice using 8 *μ*g of the epitope peptide, with a two-week interval between immunizations. Approximately, 4 *μ*g of epitope peptide emulsified in complete Freund's adjuvant was used for the first immunization, 4 *μ*g of epitope peptide emulsified in incomplete Freund's adjuvant was used for the second immunization, and 4 *μ*g of epitope peptide alone was used for the final immunization. The control group was immunized with PBS using the same procedure. The mice were anesthetized and were then challenged with the HSV-2 Sav strain three weeks after the final immunization. The immunized and sham-immunized mice were subcutaneously injected with 2 mg of progesterone (Jinsite, China) in 50 *μ*L of H_2_O per mouse to synchronize the estrus cycle at the progesterone-dominated stage. On the 5th day after the administration of progesterone, all the mice were challenged with HSV-2 Sav strain through their vagina and external genital skin. The vaginal closure membrane was ruptured with a saline-moistened cotton swab an hour prior to the virus challenge. The swab was inserted into the vagina of each mouse, twisted back and forth five times, and then removed and wiped over the external genitalia. The infected mice were examined daily for vaginal inflammation, neurological illness, and death and were then scored in grades 1–5 depending on the severity of the disease, as described in a reported paper [17]. The survival ratio of the mice was calculated daily.

### 2.10. Statistical Analysis

Data were the mean values of at least three different experiments and expressed as mean ±SD. Student's *t*-test was used to compare different data sets. The differences between groups were compared with one another using one-way ANOVA. *P* < 0.05 was considered statistically significant.

## 3. Results

### 3.1. Prediction and Identification of B-Cell Epitopes

Twenty B-cell immunodominant epitopes in glycoproteins gB2, gC2, gE2, gG2, and gI2 of HSV-2 were predicted using the DNAstar algorithm. The predicted *β*-turn, flexibility, coil regions, glycosylation sites, and antigen value of the epitopes are listed in Table 1. The antigenicity of the predicted immunodominant epitopes was detected using EIA of the antibodies to their corresponding parent glycoproteins. The epitope peptides were coated as antigens in 96-well plates, with 100 *μ*L (2.5 *μ*g/mL) in each well. The antibodies to the corresponding parent glycoproteins gB2, gC2, gE2, gG2, and gI2 (ABcam) reacted with the epitopes, respectively. The A_450_ values were measured, and the results (Table 1) show that 17 out of the 20 epitopes had various levels of antigenicity. Three epitopes, namely, gB2_468–475_, gB2_300–306_, and gC2_326–335_, were predicted to have strong antigenicities using the software algorithms, but their antigenicities were shown to be weak in the EIA tests (Table 1). Overall, combining the prediction results using the three software algorithms (Table 1) with the EIA results (Tables 1 and 2), at least five epitopes (listed in Table 1 in bold), namely, gB2_466–473_ (EQDRKPRN), gC2_216–223_ (GRTDRPSA), gE2_483–491_ (DPPERPDSP), gG2_572–579_ (EPPDDDDS), and gI2_286–295_ (CRRRYRRPRG), were confirmed as strong B cell antigenic epitopes. All conjugates of the five peptides with the carrier protein BSA could stimulate mice into producing antibodies. Moreover, the antisera to the five peptides reacted strongly with the corresponding parent glycoproteins during the EIA tests, and the peptides reacted strongly with the antibodies against the corresponding parent glycoproteins during the EIA tests (Table 2).

### 3.2. Specificity of Antipeptide Antibodies

**Figure 1:**
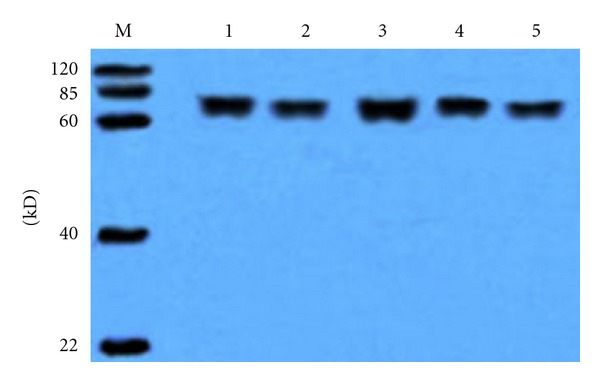
Western blotting results of the five peptides-BSA to the antibodies against the parent proteins, respectively. (1) gB2_466–473_/BSA, (2) gC2_216–223_/BSA, (3) gE2_483–491_/BSA, (4) gG2_572–579_/BSA, (5) gI2_286–295_/BSA.

**Figure 2:**
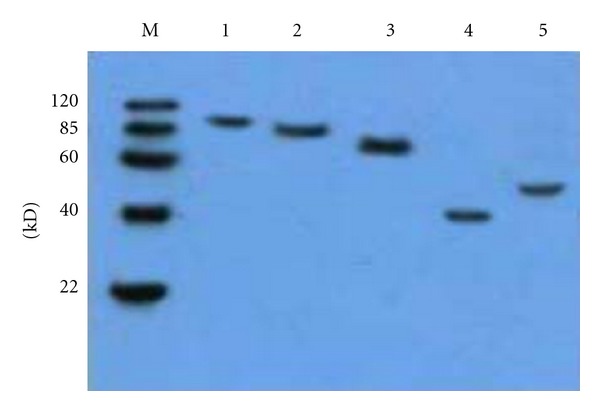
Western blotting results of the five parent proteins to the antibodies against the peptides-BSA, respectively. (1) gB2, (2) gC2, (3) gE2, (4) gG2, (5) gI2.

The specificity of the antisera to the five epitopes was examined further using Western Blot analysis. The epitope-BSA conjugates were used as antigens for detecting the antibodies to the corresponding parent proteins and as antisera to the five epitopes detected with the corresponding parent proteins gB2, gC2, gE2, gG2, and gI2. The results (Table 3, Figures 1 and 2) show that the epitope-BSA conjugates reacted specifically with the antibodies to the corresponding parent proteins without any cross-action and that the antisera to the five epitopes could react with the corresponding parent proteins.

### 3.3. Virus Neutralization Activity of the Antisera

The virus-neutralization activity of the antisera against the five epitopes was tested using the 50% plaque reduction assay. The results (Table 4) show that the antisera to the five epitopes could block HSV-2 infection. The neutralization titers of the antisera against gB2_466–473_ and gE2_483–491 _were 1 : 256, the neutralization titers of the antisera against gC2_216–223_, gG2_572–579_, and gI2_286–295_ were 1 : 128, they were essentially the same neutralization titer. On the other hand, the control sera from the PBS-vaccinated mice and the antisera to BSA did not show neutralization activities.

### 3.4. Animal Protection Experiments

**Figure 3:**
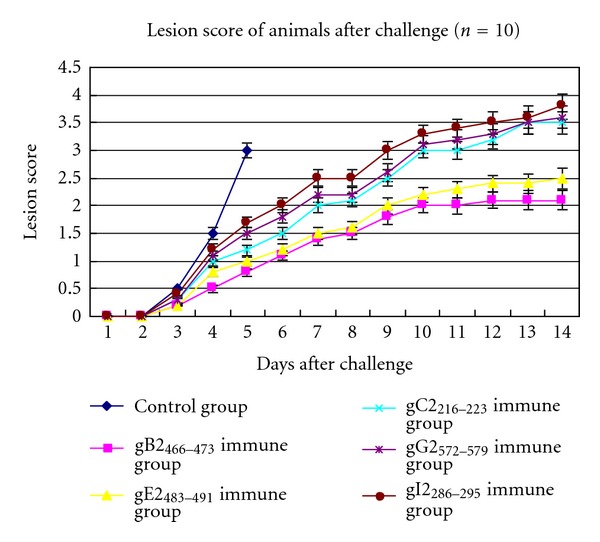
Lesion score of the immunized animals after HSV-2 challenge.

The immunized mice were intravaginally challenged with lethal dosages (5 × 10^6^ pfu, 500 LD_50_ for each mouse) of HSV-2 Sav strain. The vaginal external diseases were examined daily for inflammation, and the mean daily lesion scores were measured on the 1st to the 14th day following the challenge. The severity of the primary disease was assessed (1–5 scores) using the lesion scoring system [17]. The mice in all groups immunized with the five epitopes, respectively, were protected from death to a certain extent at a lethal dose, whereas the mice in the control group developed severe diseases and died, on the 3rd day after the challenge. The vaccinated mice showed some extended protection level, and the lesion scores of the infected mice varied among the five groups from the 4th day after the challenge (Figure 3). On the 4th day after the challenge, the mice immunized with gB2_466–473_/BSA or gE2_483–491_/BSA showed high protection level, with lesion scores of less than 1, whereas the mice immunized with gC2_216–223_/BSA, gG2_572–579_/BSA, or gI2_286–295_/BSA showed relatively low protection level, with lesion scores of greater than 1. These results indicated that the inflammation severity of the immunized mice with the epitopes significantly decreased.

**Figure 4:**
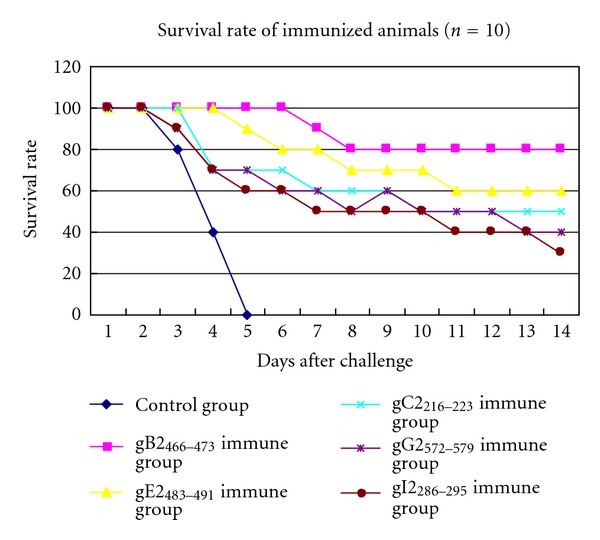
Survival rates of the immunized mice after the HSV-2 challenge.

The survival rates of the infected mice were calculated daily. The survival rates of all the epitope-immunized groups ranged from 50% to 80% (Figure 4), whereas all the PBS-vaccinated mice developed severe diseases and died between the 3rd and 5th day after the challenge. On the 8th day after the challenge, the survival rate of the gB2_466–473_-immunized mice was highest (80%), and the survival rate of the gE2_483–491_/BSA-immunized mice was 70%. Protective effect of the five epitope peptides was significantly better than the control group (student's *t*-test, *P* < 0.05), gB2_466–473_ was the best, followed by gE2_483–491_, and there were not significantly difference in the protective effect among the other three epitope peptides (student's *t*-test, *P* > 0.05). From highest to lowest, the protection rates of the five epitopes were gB2_466–473_/BSA > gE2_483–491_/BSA > gC2_216–223_/BSA > gG2_572–579_/BSA > gI2_286–295_/BSA. The results show that each of the five epitopes had a partial protection effect, but they could not completely protect the mice from infection alone.

## 4. Discussion

Predicting the antigenic, surface-located regions (B-cell epitopes) from the primary structure of the parent glycoprotein was necessary to select the epitope peptides that can stimulate mice into generating antibodies and enable these antibodies to react with the corresponding parent glycoproteins and neutralize the viral infectivity [18]. Some methods have recently been developed to predict sequential B-cell epitopes in glycoproteins (linear and conformational) [18]. In the current study, the epitopes in glycoproteins gB2, gC2, gE2, gG2, and gI2 of HSV-2 were predicted because these glycoproteins play important roles in humoral immunity. Some epitopes, such as gG2_472–479_, were selected because of their high *β*-turn probability, hydrophilicity, and predicted flexibility [19, 20]. On the other hand, some B cell epitopes, such as gE2_483–491_, were selected because of their high hydrophilicity value. Most predicted epitopes using software algorithms had good antigenicity. For example, gB2_466–473_ had high antigenicity values in the prediction algorithms and strong reaction in the EIA tests. Only few of the predicted epitopes had weak antigenicity. For example, gB2_468–475_, gB2_300–306_, and gC2_326–335_ had high antigenicity values in the prediction algorithms but had low values in the EIA tests, indicating that predicting epitopes using software algorithms is useful for selecting immunodominant epitopes.

Meanwhile, other software algorithms, such as the surface plot predictive algorithm, have also been used for selecting the linear amino acid sequence regions (B-cell epitopes) of proteins [21]. The method, which included the Chou-Fasman secondary structure, glycosylation site, and *β*-turn algorithms, was similar to the prediction algorithm in the current study. In our opinion, any prediction method is limited for selecting the epitopes in viral proteins that may elicit neutralizing antibodies, and none of the prediction algorithms is significantly better than the other methods. Therefore, B-cell antigenic epitopes from HSV-2 glycoproteins were screened in the current study using three software algorithms, namely, the Biosun, DNAstar, and Antheprot algorithms, combined with the multiparameter algorithm. The common results of the software algorithms were used as candidate epitopes of B-cell epitopes. Several examples in literature implicated that a buried region of an antigen could be immunogenic. For example, two sites in the vpI coat protein of poliovirus induced a significant neutralization response in rats and rabbits, although these regions had been shown to be deeply buried in the interior using X-ray crystallographic studies [22]. In the current study, the screened B-cell epitopes were linear antigenic regions presented on the surface of the molecule and were not conformational or discontinued epitopes.

The epitope (473–730 aa) of gB2 had been reported to have high antigenicity [22], which is consistent with the results of the current study, in which the 466–473 aa of gB2 is the immunodominant epitope of gB2. The epitope (210–230 aa) of gC2 was reported to be the immunodominant epitope [23], which is consistent with the prediction in the current study. However, the epitope (216–223 aa) of gC2 was selected in the current study because it had higher antigenicity than the epitope (126–132 aa) of gC2, as shown in the EIA results. Three epitopes, namely, 350–364 aa, 286–295 aa, and 526–539 aa of gG2, have also been reported [24], but the selected epitope (472–479 aa) of gG2 in the current study had a stronger antigenicity. In the current study, the five predicted epitopes, namely, 466–473 aa of gB2, 216–223 aa of gC2, 483–491 aa of gE2, 472–479 aa of gG2, and 286–295 aa of gI2, which have not been reported before were confirmed using EIA, Western Blot, and animal protection experiments.

The five predicted epitopes could induce mice into producing specific antibodies, and all the antisera had virus neutralization activities. Some previous studies have demonstrated that neutralizing antibodies are important for the protection against HSV-2 infection and the titer of ≥ 1 : 10 is considered to be indicative of protective immunity [25]. The mean neutralization titer of the antisera from the mice immunized with gB2_466–473_ or gE2_483–491_ was 1 : 256, and the mean neutralization titer of the antisera from the mice immunized with gC2_216–223_, gG2_572–579_, or gI2_286–29 _g was 1 : 128. These antibody titers were high enough to neutralize virus infection. Moreover, each of the five epitope peptides could partially protect the mice from HSV-2 infection. However, none of the five epitope peptides could completely protect the mice from HSV-2 infection; thus, they should be used together as a mixed immunogens to elicit stronger and more complete immunity protection.

## 5. Conclusions

Five predicted immunodominant epitope peptides, namely, gB2_466–473_, gC2_216–223_, gE2_483–491_, gG2_572–579_, and gI2_286–295_ from glycoproteins B, C, E, G, and I of HSV-2, respectively, were confirmed via experiments. The five epitope peptides showed excellent antigenicity and could stimulate mice into producing specific antibodies that could neutralize HSV-2 infection *in vitro*. The five epitope peptides can also partially protect mice from HSV-2 infection. Thus, these epitope peptides may be used for HSV-2 infection diagnosis and vaccine design.

## Figures and Tables

**Table 1:** Predicted antigenicity (antigen value) of the 20 epitopes and the EIA detection results.

Number	Predicted epitope	Surface probability	Flexibility	*β*-turn	Coil	Glycosylation site Asn-X-Thr/Ser	Predicted antigen value	EIA A_450_ value
(1)	gB2_58–75 _	5.219	44–82	69, 71	69, 70		1.588	0.361 ± 0.011
(2)	gB2_466–473 _	5.685	465–485	473	470		**2.31 **	0.987 ± 0.031
(3)	gB2_468–475 _	4.185	465–475	473	470	+	1.593	0.276 ± 0.011
(4)	gB2_300–306 _	1.984	300–306	301	301–305		2.057	0.118 ± 0.024
(5)	gC2_326–335 _	1.320	328–335	330–332	333–335	+	2.044	0.278 ± 0.011
(6)	gC2_95–105 _	1.315	95–100	93, 98	100–104		2.01	0.687 ± 0.022
(7)	gC2_161–171 _	1.948	161–170	167	164–170	+	1.736	0.689 ± 0.021
(8)	gC2_216–223 _	2.242	216–223	217, 222	219–223		**2.235 **	0.898 ± 0.030
(9)	gE2_514–522 _	3.446	514–522	519	515		2.322	0.655 ± 0.030
(10)	gE2_471–479 _	2.02	471–479	478	471–47		2.287	0.877 ± 0.031
(11)	gE2_483–491 _	2.972	484–495	488, 492	485, 493		**2.367 **	1.107 ± 0.042
(12)	gE2_385–398 _	2.701	385–398	396	385–394		2.49	0.564 ± 0.021
(13)	gG2_526–539 _	2.613	526–537	535, 539	530, 534		1.866	0.456 ± 0.019
(14)	gG2_286–295 _	1.215	289–293	290	288		1.56	0.821 ± 0.025
(15)	gG2_572–579 _	2.767	569–577	569, 574	570		**2.807 **	0.811 ± 0.031
(16)	gG2_350–364 _	1.844	350–364	351	353–358		2.124	0.992 ± 0.033
(17)	gI2_202–226 _	2.453	202–226	203, 210, 217	205	+	1.859	0.413 ± 0.011
(18)	gI2_236–253 _	2.143	236–253	242, 253	241, 244–9, 251		2.081	0.856 ± 0.026
(19)	gI2_286–295 _	4.729	287–295	287, 293			**2.764 **	1.034 ± 0.039
(20)	gI2_319–337_	3.427	319–337	323, 326, 333	321, 325, 335		2.042	0.569 ± 0.021

C								0.121 ± 0.011

**Table 2:** Antigenicity and specificity analysis of the five epitopes using EIA.

Number	Antigen (peptides-BSA)	Antibody to the proteins (titer)	OD value (450 nm)	Antigen (proteins)	Antibody to peptides-BSA (titer)	EIA A_450_ value
(1)	gB2_466–473_/BSA	gB2 (1 : 10 000)	0.977 ± 0.039	gB2	gB2_466–473_/BSA (1 : 5000)	1.099 ± 0.037
(2)	gC2_216–223_/BSA	gC2 (1 : 10 000)	0.899 ± 0.031	gC2	gC2_216–223_/BSA (1 : 5000)	1.102 ± 0.040
(3)	gE2_483–491_/BSA	gE2 (1 : 10 000)	1.102 ± 0.040	gE2	gE2_483–491_/BSA (1 : 5000)	0.893 ± 0.029
(4)	gG2_572–579_/BSA	gG2 (1 : 10 000)	0.882 ± 0.011	gG2	gG2_572–579_/BSA (1 : 5000)	0.986 ± 0.031
(5)	gI2_286–295_/BSA	gI2 (1 : 10 000)	0.792 ± 0.029	gI2	gI2_286–295_/BSA (1 : 5000)	0.876 ± 0.031
(6)	BSA	All the proteins^#^ (1 : 10 000)	<0.2000	All the proteins	BSA** (1 : 5000)	<0.2000

^#^: BSA did not react with all the antisera to the five proteins.

**: The five proteins did not react with all the antisera to BSA.

**Table 3:** Antigenicity and specificity detection of the five peptides using Western Blot analysis.

Number	Antigen (peptides-BSA)	Antibody to the proteins (titer)	Western blotting	Antigen (proteins)	Antibody to peptides-BSA (titer)	Western blotting
(1)	gB2_466–473_/BSA	gB2 (1 : 10 000)	++*	gB2	gB2_466–473_/BSA (1 : 5000)	++
(2)	gC2_216–223_/BSA	gC2 (1 : 10 000)	++	gC2	gC2_216–223_/BSA (1 : 5000)	++
(3)	gE2_483–491_/BSA	gE2 (1 : 10 000)	+++	gE2	gE2_483–491_/BSA (1 : 5000)	+++
(4)	gG2_572–579_/BSA	gG2 (1 : 10 000)	++	gG2	gG2_572–579_/BSA (1 : 5000)	++
(5)	gI2_286–295_/BSA	gI2 (1 : 10 000)	++	gI2	gI2_286–295_/BSA (1 : 5000)	++
(6)	BSA	All the proteins^#^ (1 : 10 000)	—	All the proteins	BSA** (1 : 5000)	—

*: +++, strong reaction; ++, good reaction; +, moderate reaction; —, no reaction.

^#^: BSA did not react with all the antisera to the five proteins.

**: The five proteins did not react with the antisera to BSA.

**Table 4:** Neutralization activity of the antisera to the five epitopes.

Number	Antibody to epitopes	Virus (HSV-2)	50% neutralization antibody titer
(1)	gB2_466–473_/BSA	5 × 10^6^ pfu, 500 LD_50_	1 : 256
(2)	gC2_216–223_/BSA		1 : 128
(3)	gE2_483–491_/BSA		1 : 256
(4)	gG2_572–579_/BSA		1 : 128
(5)	gI2_286–295_/BSA		1 : 128
(6)	BSA		0
